# Functional Characterization of a Novel Family of Acetylcholine-Gated Chloride Channels in *Schistosoma mansoni*

**DOI:** 10.1371/journal.ppat.1004181

**Published:** 2014-06-12

**Authors:** Kevin MacDonald, Samuel Buxton, Michael J. Kimber, Tim A. Day, Alan P. Robertson, Paula Ribeiro

**Affiliations:** 1 Institute of Parasitology, McGill University, Macdonald Campus, Ste. Anne de Bellevue, Quebec, Canada; 2 Department of Biomedical Sciences, Iowa State University, Ames, Iowa, United States of America; University of Pennsylvania, United States of America

## Abstract

Acetylcholine is the canonical excitatory neurotransmitter of the mammalian neuromuscular system. However, in the trematode parasite *Schistosoma mansoni*, cholinergic stimulation leads to muscle relaxation and a flaccid paralysis, suggesting an inhibitory mode of action. Information about the pharmacological mechanism of this inhibition is lacking. Here, we used a combination of techniques to assess the role of cholinergic receptors in schistosome motor function. The neuromuscular effects of acetylcholine are typically mediated by gated cation channels of the nicotinic receptor (nAChR) family. Bioinformatics analyses identified numerous nAChR subunits in the *S. mansoni* genome but, interestingly, nearly half of these subunits carried a motif normally associated with chloride-selectivity. These putative schistosome acetylcholine-gated chloride channels (SmACCs) are evolutionarily divergent from those of nematodes and form a unique clade within the larger family of nAChRs. Pharmacological and RNA interference (RNAi) behavioral screens were used to assess the role of the SmACCs in larval motor function. Treatment with antagonists produced the same effect as RNAi suppression of SmACCs; both led to a hypermotile phenotype consistent with abrogation of an inhibitory neuromuscular mediator. Antibodies were then generated against two of the SmACCs for use in immunolocalization studies. SmACC-1 and SmACC-2 localize to regions of the peripheral nervous system that innervate the body wall muscles, yet neither appears to be expressed directly on the musculature. One gene, SmACC-1, was expressed in HEK-293 cells and characterized using an iodide flux assay. The results indicate that SmACC-1 formed a functional homomeric chloride channel and was activated selectively by a panel of cholinergic agonists. The results described in this study identify a novel clade of nicotinic chloride channels that act as inhibitory modulators of schistosome neuromuscular function. Additionally, the iodide flux assay used to characterize SmACC-1 represents a new high-throughput tool for drug screening against these unique parasite ion channels.

## Author Summary

Schistosomiasis is a widespread, chronic disease affecting over 200 million people in developing countries. Currently, there is no vaccine available and treatment depends on the use of a single drug, praziquantel. Reports of reduced praziquantel efficacy, as well as its ineffectiveness against larval schistosomula highlight the need to develop new therapeutics. Interference with schistosome motor function provides a promising therapeutic target due to its importance in a variety of essential biological processes. The cholinergic system has been shown previously to be a major modulator of parasite motility. In this study, we have described a novel clade of schistosome acetylcholine-gated chloride channels (SmACCs) that act as inhibitory modulators of this pathway. Our results suggest that these receptors are absent in the human host and indirectly modulate inhibitory neuromuscular responses, making them an attractive drug-target. We have also validated a new functional assay to characterize these receptors, which may be modified for future use as a high-throughput drug screening method for parasite chloride channels.

## Introduction

Flatworms of the genus *Schistosoma* are the causative agents of the debilitating parasitic infection schistosomiasis, afflicting over 230 million people in 74 endemic countries [1]. The majority of human schistosomiasis can be attributed to three species- *S. mansoni*, *S. japonicum* and *S. haematobium*- which cause a wide spectrum of chronic pathology, including hepatosplenomegaly, portal hypertension and squamous cell carcinoma [1]. Currently, praziquantel (PZQ) is the only drug used to treat schistosomiasis and there is no vaccine available. Widespread and exclusive use of PZQ has led to concerns of emerging drug resistance. Laboratory strains of PZQ-resistant *S. mansoni* have been successfully generated and there are now several reports of reduced PZQ cure rates in the field [2], [3]. Moreover, PZQ is ineffective in killing larval schistosomulae [4]. The stage-limited efficacy of PZQ and looming prospect of drug resistance signal the importance of exploring novel therapeutic targets for the treatment of schistosomiasis.

An area of interest for the treatment of helminth parasites is the neuromuscular system, which is targeted by the majority of currently approved and marketed anthelminthics [5]. Inhibition of neuromuscular activity provides two modes of treatment. First, motor inhibition may interfere with parasite maturation, which is closely tied with migration during the larval stage [6]. Second, a loss of muscle function would disrupt essential activities, including attachment to the host, feeding, mating and others [7], ultimately causing the parasite to be eliminated from the host. The cholinergic system has proved especially successful as a neuromuscular anthelminthic target. Common antinematodal drugs such as levamisole, pyrantel and monepantel [5], [8], and the antischistosomal drug, metrifonate [9], all disrupt neuromuscular signaling by interacting with proteins of the worm's cholinergic system.

Acetylcholine (ACh) is an important neurotransmitter in both vertebrate and invertebrate species. The neuromuscular effects of ACh are typically mediated by postsynaptic nicotinic acetylcholine receptors (nAChRs), so named because of their high-affinity for nicotine. Structurally, nAChRs are members of the Cys-loop ligand-gated ion channel (LGIC) superfamily. They form homo- and heteropentameric structures, which are organized in a barrel shape around a central ion-selective pore [10]. Vertebrate nAChRs are invariably cation-selective (Na^+^, Ca^2+^, K^+^) and mediate excitatory responses. Invertebrates, on the other hand, have both cation and anion-selective (Cl^−^) ACh-gated channels. The latter mediate Cl^−^ - driven membrane hyperpolarization and therefore are believed to play a role in inhibitory responses to ACh. One example of these unique invertebrate receptors is the acetylcholine-gated chloride channel (ACC) of the snail, *Lymnaea*, which is structurally related to nAChRs, yet is selective for chloride ions [11]. In addition, nematodes have an unusual type of ACC, which is a functional acetylcholine-gated chloride channel but is more closely related to other chloride channels (GABA and glycine receptors) than nAChRs [12]–[13]. A defining feature of the ACCs is the presence of a Pro-Ala motif in the pore-lining M2 domains of the constituent subunits. This motif, which has been shown to confer anion-selectivity to other LGICs, replaces a Glu residue normally found in the cation-selective channels [14].

ACCs have not been identified in any of the flatworms, free-living or parasitic. However, there is experimental evidence supporting an inhibitory role for ACh in the parasites, which could be mediated by this type of receptor. Early studies in the 1960s observed that addition of exogenous cholinergic agonists to parasite cultures caused flaccid paralysis of adult trematodes and cestodes [15]–[16]. Flaccid paralysis indicates muscular relaxation and is in direct contradiction to the excitatory response of tonic contraction expected from cholinergic stimulation. Later research established a causal relationship between activation of a nicotinic-like receptor in *S. mansoni* muscle fibers and the flaccid paralysis caused by ACh in whole worms [17]. However, this work was performed in the pre-genomic era and no attempt was made to clone or characterize the receptors involved. More recently, the publication of the *S. mansoni* genome [18] has provided cause to revisit the unusual inhibitory activity of ACh in schistosomes. Several candidate genes have been annotated as nAChR subunits [18]–[19] and the present work aims to confirm the presence of and functionally characterize cholinergic chloride channels in *S. mansoni*.

One strategy that has been used for assessing the therapeutic value of candidate genes in parasites, particularly helminths, is RNA interference (RNAi) [20]–[22]. A strength of this reverse genetics strategy is the ability to screen living animals for phenotypic and behavioral changes as a result of abrogation of a particular gene's function, as demonstrated by the large-scale screens in the free-living platyhelminth cousins of schistosomes, the planarians [23]. The RNAi pathway is conserved in *S. mansoni* [20]–[21] and has previously been used to probe the neuropeptidergic system of the parasite [24] and, more recently, the serotonergic system as well [25]. However, the effects of silencing other important neuroactive pathways, such as the cholinergic system, are not known.

Here we describe a novel clade of anion-selective nAChR subunits (SmACCs) that appear to be invertebrate-specific. The ion channels formed by these subunits play an inhibitory role in the neuromuscular activity of the parasite, as suggested by the results of RNAi and pharmacological behavioral assays, their tissue distribution and pharmacological properties.

## Materials and Methods

### Parasites

A Puerto Rican strain of *S. mansoni*-infected *Biomphalaria glabrata* snails were kindly provided by Dr. Fred Lewis (Biomedical Research Institute and BEI Resources, MD, USA) and used for all experiments. To obtain larval schistosomula, 6–8 week-old snails were exposed to bright light for 2 hours at room temperature. The resulting cercarial suspension was mechanically transformed *in vitro* by vortexing, washed twice with Opti-MEM (Gibco) containing 0.25 µg/ml fungizone, 100 µg/ml streptomycin and 100 units/ml penicillin and cultured in Opti-MEM/antibiotics supplemented with 6%FBS (Gibco) [26]. To obtain adult worms, 40-day old female CD1 mice were injected intraperitoneally with 250 mechanically transformed schistosomula [26]. After 8 weeks, adult worms were collected by perfusion of the mouse hepatic portal vein and mesenteric venules, as previously described [26]. Animal procedures were reviewed and approved by the Facility Animal Care Committee of McGill University (Protocol No. 3346) and were conducted in accordance with the guidelines of the Canadian Council on Animal Care.

### Bioinformatics

To generate a target list of putative nicotinic acetylcholine receptor (nAChR) subunits, the *S. mansoni* Genome Database was searched using the keywords “nicotinic” and “acetylcholine receptor” [18]–[19]. A BLASTp homology search was also performed using the *Torpedo* nAChR (AAA96704.1) as a query. The resulting list of nAChR subunit sequences was used as a query against the general NCBI protein database and aligned with other Cys-loop receptor superfamily proteins by CLUSTALX [27]. The alignments were analyzed manually to identify the presence of the vicinal C motif, indicative of nAChR α-subunits, and key amino acids involved in ion-selectivity. Phylogenetic trees were built in PHYLIP using the neighbor-joining method and bootstrapped with 1,000 replicates [28]. Trees were visualized and annotated using FigTree3.0 [29] and manually inspected to ensure that bootstrap values for each node were above a 70% threshold.

### siRNA Design and Synthesis

Five putative nAChR subunits were targeted by RNA interference (RNAi): Smp_157790, Smp_037960, Smp_132070, Smp_176310 (SmACC-1) and Smp_142690 (SmACC-2). For each target sequence, we amplified a unique 200–300 bp PCR fragment by RT-PCR. Total RNA was extracted from pooled adult male and female *S. mansoni,* using the RNeasy Micro Kit (Qiagen) and reverse-transcribed with MML-V (Invitrogen) and Oligo-dT (Invitrogen). PCR amplification was performed with a proofreading Phusion High Fidelity Polymerase (New England Biolabs), according to standard protocols. PCR primers (Table S2) were designed using Oligo6.2 [30] and the unique fragment sequences were identified by BLAST analysis. Amplicons were ligated to the pJET1.2 Blunt Vector (Fermentas) and verified by sequencing of multiple clones. For synthesis of double-stranded RNAs (dsRNA), the T7 promoter sequence (5′-TAATACGACTCACTATAGGGAGA-3′) was added to both ends of each target fragment by PCR. Long dsRNAs were generated from the resulting T7-flanked PCR products by in vitro transcription of both DNA strands, using the MegaScript T7 Transcription Kit (Ambion), according to the kit protocol. The dsRNAs were subsequently digested with RNAseIII, using the Silencer siRNA Kit (Ambion), to generate a mixture of siRNAs for each target. The siRNA was quantitated and assessed for purity using a Nanodrop ND1000 spectrophotometer.

### Transfection of Schistosomula and Motility Assays

Larval schistosomula were obtained by the standard protocol (see above) with some modification. After the final wash, freshly transformed schistosomula were re-suspended in Opti-MEM without antibiotics or FBS and plated at a concentration of 100 animals/well in a 24-well plate. Animals were transfected using siPORT NEO FX Transfection Agent (Ambion) and either an irrelevant scrambled siRNA (Ambion) or nAChR subunit-specific siRNA at a final concentration of 50 nM. Transfections were performed blind to rule out selection bias during analysis. Opti-MEM containing antibiotics and supplemented with 6%FBS was added to transfected schistosomula 24 hours post-treatment. A previously described larval motility assay was performed 6 days post-transfection [31]. Briefly, schistosomula were filmed for 45s using a Nikon SMZ1500 microscope equipped with a digital video camera (QICAM Fast 1394, mono 12 bit, QImaging) and SimplePCI version 5.2 (Compix Inc.) software. Three distinct fields were recorded for each well. ImageJ (version 1.41, NIH, USA) software was then used to quantitate worm motility using the Fit Ellipse algorithm in ImageJ, as described [25]. The data shown here are derived from three independent experiments in which a minimum of 12 animals was measured per experiment. Pharmacological motility assays were carried out with 6-day old schistosomulae in the same manner, but without the transfection with siRNA. Baseline measurements of schistosomula motility were recorded prior to drug addition. Compounds of interest (arecoline, nicotine, mecamylamine, D-tubocurarine) were subsequently added at a final concentration of 100 µM and larval motility was measured again after 5 minutes exposure. Viability of drug-treated and siRNA-treated schistosomula was routinely monitored by a dye exclusion assay, according to the method of Gold [32].

### Real-Time Quantitative PCR

Six-day old siRNA-treated schistosomula were washed twice with 1X PBS, re-suspended in the lysis buffer provided with the RNEasy Micro RNA Extraction Kit (Qiagen) and sonicated with 6 pulses of 10 s each. Total RNA was then extracted from the lysate following the manufacturer's instructions. RNA was quantified and assessed for purity using a Nanodrop ND1000 spectrophotometer. 100 ng total RNA was used for each 20 µl MML-V (Invitrogen) reverse transcription (RT) reaction, which was performed according to standard protocols. A negative control lacking reverse transcriptase was also prepared in order to rule out contamination with genomic DNA. Quantitative real-time PCR (qPCR) was performed using the Platinum SYBR Green qPCR SuperMix-UDG kit (Invitrogen) in a 25 µl reaction volume. Primers located in a unique region of each gene and separate from those regions used to generate siRNA were designed using Oligo6.2 and may be found in Table S2. Primers targeting the housekeeping gene glyceraldehyde 3-phosphate dehydrogenase (GAPDH, Accession #M92359) were used as an internal control and are as follows: forward 5′-GTTGATCTGACATGTAGGTTAG- 3′ and reverse 5′-ACTAATTTCACGAAGTTGTTG-3′. Primer validation curves were generated to ensure similar efficiency of target and housekeeping gene amplification. Cycling conditions were as follows: 50°C/2 min, 95°C/2 min, followed by 50 cycles of 94°C/15 s, 57°C/30 s, 72°C/30 s. Cycle threshold (Ct) values were normalized to GAPDH and then compared to the scrambled siRNA control, as well as an off-target gene (another nAChR subunit) to ensure transcript-specific silencing. All expression data was analyzed using the comparative ΔΔCt method [33] and was generated from three separate experiments done in triplicate.

### Cloning of Full Length SmACC-1 and SmACC-2

Two putative anion-selective subunit sequences, Smp_176310 (SmACC-1) and Smp_142690 (SmACC-2) were chosen for further study and cloned by conventional RT-PCR (see above) using primers targeting the beginning and end of each cDNA. For SmACC-1 we used primers: forward 5′-ATGGATCTAATATACTTG-3′ and reverse: 5′-TTAGGTAGTTTCTTCTG-3′. PCR conditions were as follows: 98°C/30 s, 30 cycles of 98°C/10 s, 55°C/60 s, 72°C/90 s and final extension of 72°C/5 min. In the case of SmACC-2, the full-length cDNA was amplified with primers 5′-ATGGAAAAATCACTTATTCG-3′ (forward) and 5′-TTATTGTAGATCAACTACG-3′ (reverse), using the following cycling conditions: 98°C/30 s, 30 cycles of 98°C/10 s, 54°C/60 s, 72°C/60 s and a final extension of 72°C/5 min. The 5′ end of SmACC-2 was further verified by 5′ RACE (rapid amplification of cDNA ends), using a commercial kit (Invitrogen) and a gene-specific primer for the reverse transcription [5′-GCAGGTACATAATCTGAG-3′], according to manufacturer's instructions. All PCR products were ligated to the pJet1.2 Blunt cloning vector (Thermo Scientific) and verified by DNA sequencing of at least two independent clones.

### Antibody Production

Peptide-derived polyclonal antibodies were generated in rabbits against subunits SmACC-1 and SmACC-2 (21^st^ Century Biochemicals – Marlborough, MA). Animals were injected with a mixture of two specific peptides per target. For SmACC-1, the two peptides 1(NAKVNRFGKPHGNKFC) and 2(CSKKALSAANAKWNSPLQY) are located in the third intracellular loop of the protein. For SmACC-2, peptide 1 (TDGEAERHIRHEDRVHQLRSVC) and peptide 2 (LQNINMKQIKLEYKNSLGC) are located at the N- and C-terminal ends, respectively. All peptides were conjugated to the carrier protein ovalbumin and were BLASTed against the *S. mansoni* genome database and the NCBI general database to ensure specificity. Whole antisera were tested for specificity and titer against both immunogenic peptides by ELISA. The anti-nAChR-specific IgG fractions were affinity-purified, using beads that were covalently attached to a mixture of the two peptide antigens added in equal amounts. Peptide conjugation to the beads and subsequent affinity purification were performed with the Pierce Sulfolink Kit for Peptides (Thermo Scientific), according to manufacturer's instructions. ELISA was performed to determine the titer of affinity-purified antibody fractions. Protein was quantified by the Bradford assay, using a commercial kit (BioRad, USA). A mouse monoclonal anti-FLAG M2 antibody was purchased from Sigma-Aldrich.

### Confocal Microscopy

Parasites were prepared for confocal microscopy according to previously described protocols [34], [35]. Briefly, 6-day old *in-vitro*-transformed schistosomula or freshly collected adult worms were washed two times in 1X PBS and fixed in 4%PFA for 4 hours at 4°C. Parasites were washed twice, each for 5 minutes in 1X PBS containing 100 µM glycine and then permeabilized with 1%SDS in 1X PBS for 25 minutes [36]. After permeabilization, animals were incubated overnight at 4°C in antibody diluent (AbD) containing 0.1%Tween-20, 1% BSA in PBS to block non-specific binding. After 3 washes of 10 minutes each in the AbD, animals were then incubated with affinity-purified anti-SmACC-1 or anti-SmACC-2 (1∶100) for three days at 4°C. Samples were then washed 3 times in AbD and incubated in secondary antibody (1∶1000) conjugated to Alexa Fluor 488 or 594 (Invitrogen, USA). In some experiments, tetramethylrhodamine B isothiocyanate (TRITC)-conjugated phalloidin (200 µg/ml) was added with secondary antibody and used to visualize the musculature. Secondary antibody incubation lasted for 2 days and animals were again washed three times before mounting for microscopy. Slides were examined using a Zeiss LSM710 confocal microscope (Carl Zeiss Inc., Canada) equipped with the Zeiss Zen 2010 software package. The lasers used for image acquisition were an Argon 488 nm and a HeNe 594 nm, with the filter sets adjusted to minimize bleed-through due to spectral overlap. Negative control slides were prepared by incubating samples in pre-immune serum, secondary antibody only (primary antibody was omitted) or primary antibody preadsorbed with 0.5 mg/mL of mixed peptide antigen (0.25 mg/ml of each peptide). At least 5 independent samples were examined for each peptide-derived antibody.

### Western Blot Analysis

Membrane-enriched protein fractions were extracted from adult *S. mansoni* using the ProteoExtract Native Membrane Protein Extraction Kit (Calbiochem, USA) and following the manufacturer's instructions. Protein was quantified by the Bradford Assay (BioRad, USA) and used for SDS-PAGE and Western blot analysis. Approximately 20 µg of membrane extract was loaded on a 4–12% Tris-Glycine gel (Invitrogen, USA) and resolved by SDS-PAGE, then transferred to a PVDF membrane (Millipore, USA). A standard Western blot protocol was followed to visualize proteins. Primary antibodies used were peptide-purified anti-SmACC-1 or anti-SmACC-2 (both 1∶1000). Secondary antibody (1∶5000) was goat-anti-rabbit conjugated to horseradish peroxidase (Invitrogen, USA). Membranes were also probed with peptide antigen-preadsorbed primary antibody (1∶1000) as a negative control.

### Heterologous Expression and Functional Characterization of SmACC-1 in HEK-293 Cells

For mammalian expression studies, a human codon-optimized construct of SmACC-1 was synthesized (Genescript, USA) and inserted into the pCi-Neo (Promega) expression vector, using *NheI* and *SmaI* restriction sites. A C-terminal FLAG tag was also included in the SmACC-Neo construct to aid in the monitoring of expression. HEK-293 cells were grown to 50% confluence in Dulbecco's Modified Essential Media (DMEM) supplemented with 20 mM HEPES and 10% heat inactivated fetal calf serum. Cells were transiently transfected with the humanized SmACC-1 construct or empty vector, using XtremeGENE 9 transfection reagent (Roche), as recommended by the manufacturer. 24 hours post-transfection, cells were transduced with Premo Halide Sensor (Invitrogen), a halide-sensitive fluorescent indicator used to assess ligand-gated chloride channel function [37]–[38]. Following transduction, cells were incubated at 37°C, 5% CO_2_ overnight and seeded onto a 96-well plate at a density of 50,000 cells per well. After an 8 hour incubation at 37°C, 5% CO_2_, the cells were equilibrated with iodide assay buffer provided with the Premo Halide Sensor assay kit for at least 30 minutes at 37°C in the reading chamber of a FlexStation II scanning fluorometer (Molecular Devices). YFP fluorescence was measured for 10 s before and up to 2 minutes after the addition of test agonists. Agonists were added at a final concentration of 100 µM, or as indicated, in a total sample volume of 200 µl. Water was used as a vehicle-only negative control. Antagonist assays were performed the same way, except the cells were pre-incubated with 100 µM cholinergic antagonist (mecamylamine, D-tubocurarine, atropine) for 30 min at 37°C prior to addition of 100 µM nicotine. Receptor activity was calculated by measuring the reduction in YFP fluorescence (YFP quench) due to iodide influx over the time of measurement. Briefly, a fluorescence measurement was taken 10 s after the addition of drug (Relative fluorescence (RF)_initial_) and again after a period of 120 s (RF_final_). The RF_final_ was subtracted from the RF_initial_ to generate ΔRF. ΔRF was then divided by the RF_initial_ and multiplied by 100, resulting in a measurement of %YFP quench, as described [38]. Readings were normalized to water-treated controls and reported as Fold-Change in YFP Quench [39]. Receptor activation was also calculated by the linear-regression slope method [40] with similar results. The minimum quench threshold for all experiments was set at zero [41]. Dose response curves were fitted using the non-linear regression function of Prism 6 software (Graphpad Software, USA). Student's t-tests were performed to determine statistically significant differences at P<0.05.

### Other Methods

Calcium assays were performed using the Calcium 4 FLIPR Assay Kit (Molecular Devices, USA) with a FlexStation II fluorometer (Molecular Devices), according to the kit protocol and as described previously [42]. Briefly, HEK-293 cells expressing SmACC-1 were preloaded with a cell-permeable fluorescent calcium indicator 48 hr post-transfection, as per the kit protocol, and treated with 100 µM nicotine, 100 µM acetylcholine or water vehicle. The concentration of calcium in the extracellular medium was ≈2 mM. Intracellular calcium was measured before addition of agonist to obtain a baseline and immediately following agonist addition at 1.52 s intervals for a total of 120 s. Calcium responses were calculated as peak fluorescence levels after subtraction of the baseline, as described [42], and experiments were repeated twice (two independent transfections), each with six replicates. *In situ* immunofluorescence assays in transfected HEK-293 cells were performed according to standard protocols, using either affinity-purified anti-SmACC-1 antibody (1∶500) or a commercial monoclonal anti-FLAG (M2) antibody, as described previously [43].

## Results

### Identification of Acetylcholine-Gated Chloride Channel Subunits in *S. mansoni (SmACCs)*

**Figure 1:**
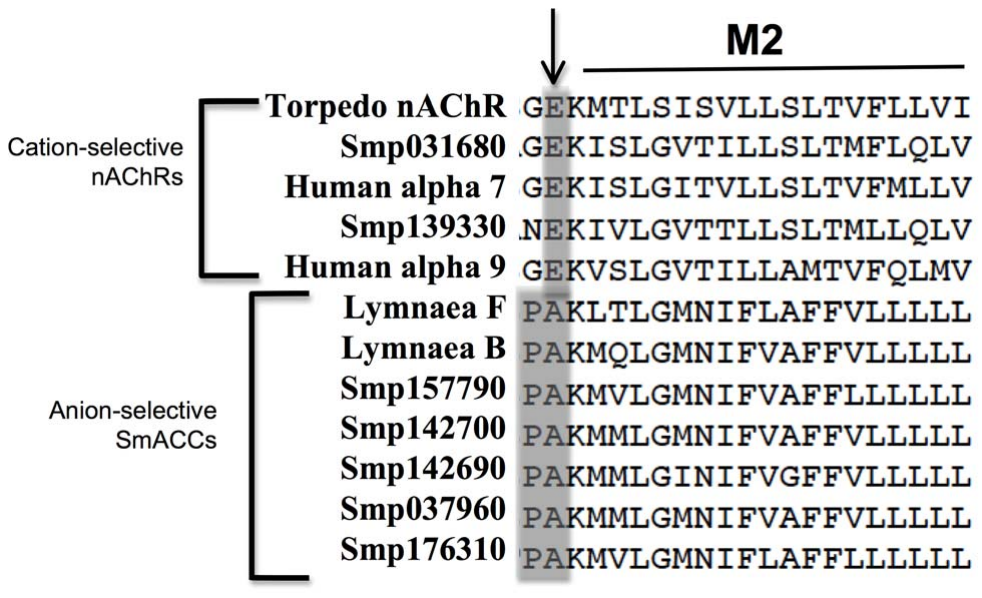
Predicted ion-selectivity of putative *S. mansoni* nAChRs. A structural alignment of human, *Lymnaea* and *S. mansoni* nAChR subunits was generated using the *Torpedo* nAChR structure (PDB Accession # 2BG9) as a template. The M1-M2 linker region, shown here, is a key determinant of ion-selectivity in Cys-loop ligand gated ion channels. A glutamate residue (arrow) confers cation-selectivity and is present in all vertebrate subunits, as well as two of the *S. mansoni* subunits. The remaining schistosome and snail subunits display a Pro-Ala motif in this position, suggesting anion-selectivity. The two subunits described in this study are identified as *S. mansoni* acetylcholine-gated chloride channels SmACC-1 and SmACC-2. Other *S. mansoni* subunits are identified by their “Smp” designation obtained from the *S. mansoni* Genome Database (*S. mansoni* GeneDB). The corresponding GenBank accession numbers are listed in Table S1.

A combination of BLAST and keyword searches were used to generate a list of potential nAChR subunits in the genome database of *S. mansoni* [18]. In total, nine putative receptor subunits were identified. All sequences were predicted to have the defining features of a nAChR subunit, including a Cys-loop motif and four transmembrane domains [44] and all subunit genes identified are predicted to contain full-length coding sequences. A structural alignment of the putative schistosome nAChR subunits with two previously characterized human nAChR alpha subunits, the *Lymnaea* nicotinic chloride channels and the crystal structure of the *Torpedo* nAChR suggests the presence of both cation and anion-selective schistosome nAChR subunits. Figure 1 shows the M2 domain of the structural alignment in which the *Torpedo*, human and two of the schistosome receptor subunits contain a conserved glutamate at the M2 interface, which is the hallmark of cation-selective Cys-loop channels. In contrast, the remaining schistosome nAChR subunits, including SmACC-1 (Smp_176310) and SmACC-2 (Smp_142690) and the *Lymnaea* subunits display a Pro-Ala motif at this position. The Pro-Ala motif is associated with anion-selectivity in Cys-loop receptors [14]. Previous mutagenesis studies have shown that replacing the M2 glutamate of a vertebrate nAChR with Pro-Ala is sufficient to convert the ion-selectivity of the channel from cationic to anionic [45, 46, see 47 for review].

**Figure 2:**
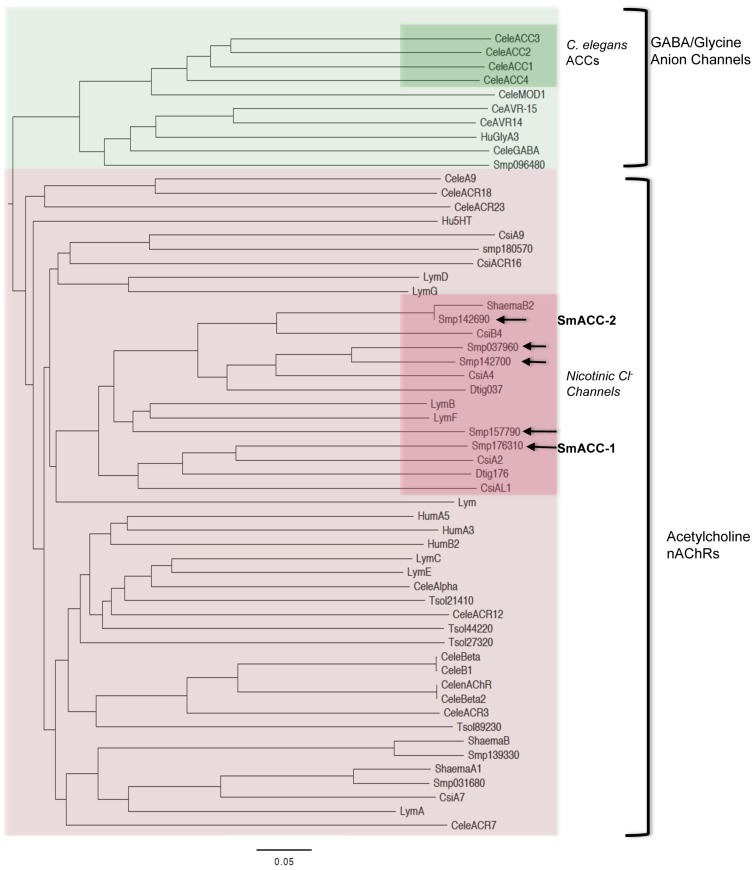
Phylogenetic analysis of cys-loop ion channel subunits. A bootstrapped, neighbor-joining tree was constructed in PHYLIP from a CLUSTALX alignment of vertebrate and invertebrate Cys-loop superfamily receptor subunits. The tree is midpoint-rooted and was visualized using FigTree 3.0. Only nodes supported by bootstrap values of 70% or higher are shown. Two distinct groups of receptors can be seen, the γ-aminobutyric acid (GABA)/glycine-like anion channels and the nicotinic acetylcholine receptors (nAChRs). The *C. elegans* acetylcholine-gated chloride channels (ACC) form a distinct clade within the larger group of GABA/glycine anion channels (green inset). In contrast the predicted *Schistosoma* acetylcholine-gated chloride channels (SmACCs) align with cholinergic nicotinic nAChRs, suggesting divergent evolutionary paths. The SmACCs described here are indicated by arrows and they constitute a separate clade in the nAChR tree along with putative homologs from flatworms *Dugesia (Dtig)*, *Clonorchis (Cs) and S. haematobium*, as well as the snail *Lymnaea (Lym)*. Accession numbers for sequences used in the alignment are listed in Table S1.

The predicted schistosome nAChRs were then aligned with cation and anion-selective Cys-loop receptor subunits from other representative vertebrate and invertebrate species, including the acetylcholine-gated chloride channel (ACC) subunits from *C. elegans* [12]. A phylogenetic tree of the alignment (Figure 2) shows the unique clade formed by the Pro-Ala motif-containing schistosome nAChR subunits is located firmly in the larger group of cation-selective nAChR subunits. Also present in this clade are the nicotinic chloride channel subunits of the snail *Lymnaea* [11] and putative homologs from fellow flatworms *Clonorchis* and *Dugesia*. This is in contrast to the *C. elegans* ACC subunits, which group more closely to the anion-selective GABA/glycine receptors and have low affinity for nicotine [12]. Thus, the nAChR subunits in schistosomes are all structurally related to cation-selective nicotinic receptors but those carrying the Pro-Ala motif appear to have diverged and may have acquired selectivity for anions. The structural relationship of the schistosome sequences to known chloride-selective nAChRs of *Lymnaea* reinforces the notion that these are nicotinic anion channels. Moreover, the presence of putative homologs in closely related flatworms and their apparent absence in host species indicate that these receptors may be good targets for broad-spectrum antiparasitics.

Two of the predicted anion-selective subunits, SmACC-1 and SmACC-2 were selected for full-length cloning. SmACC-1 contains a predicted ORF of 2415 bp distributed over 9 exons, encoding a protein of 92 kDa. SmACC-1 contains an N-terminal signal peptide and an N-terminal double cysteine motif (YxCC) that is the defining characteristic of nAChR alpha-type subunits [48]. Full-length SmACC-1 was successfully amplified by PCR and sequencing of multiple SmACC-1 clones verified the predicted ORF (GenBank accession # KF694748). The coding sequence of SmACC-2 was predicted to be 2745 bp. However, further sequence analysis by BLAST predicted a large (∼1 kb) N-terminal nucleotide-binding domain (NBD), a feature not normally present in Cys-loop receptors. This excess sequence may have been a result of the concatenation of two distinct proteins during annotation. To identify the correct start codon of SmACC-2, 5′RACE experiments were performed and an alternative start site downstream of the predicted start codon was identified, removing the NBD sequence. New PCR primers were designed and full-length SmACC-2 was amplified, resulting in a product of 1528 bp and a corresponding protein of 60 kDa (GenBank accession # KF694749). The new SmACC-2 coding sequence was in frame with the predicted ORF and retained both its Cys-loop and transmembrane domains but does not contain a signal peptide. SmACC-2 also lacks the vicinal cysteine motif, suggesting that it is a non-alpha-type nAChR subunit.

### Schistosome nAChRs Act as Inhibitory Modulators of Motor Function

**Figure 3:**
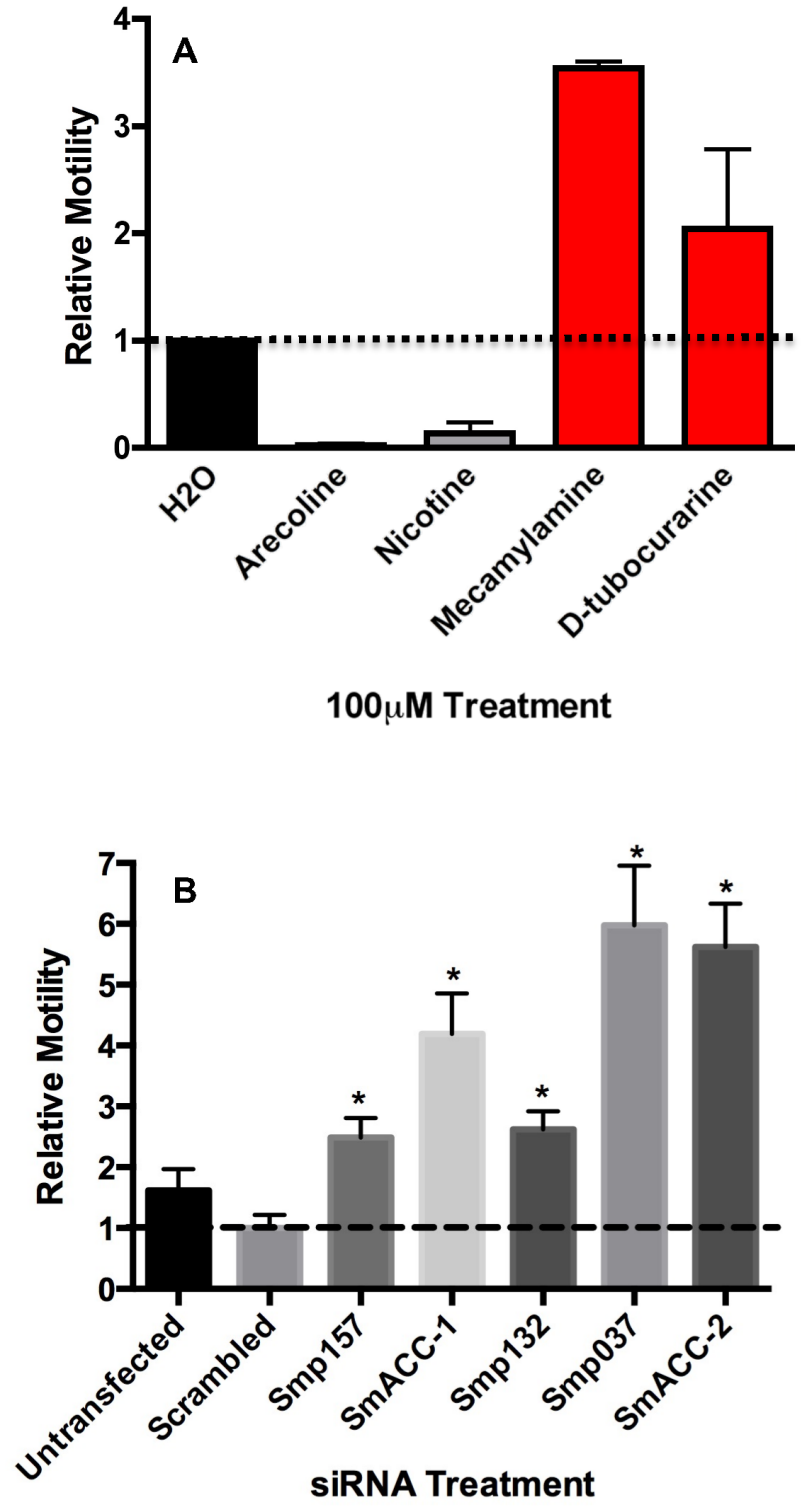
Pharmacological and RNAi behavioral assays in schistosomula. (A) Relative motility of 6-day old larvae was measured before and 5 minutes after the addition of cholinergic compounds, each at 100 µM. Data were normalized to baseline motility measured before drug addition. The data are the means and SEM of three independent experiments, each containing at least 12 animals. (B) Freshly transformed schistosomula were transfected with 50 nM irrelevant (scrambled) siRNA or 50 nM siRNA targeting a specific subunit. The following subunits were targeted in this study (refer to Table S1 for Accession numbers): Smp_157, Smp_157790; Smp_132, Smp_132070; Smp_037, Smp_037960; SmACC-1, Smp_176310; SmACC-2, Smp_142690. Larval motility was measured 6 days post-transfection and normalized relative to untransfected larvae cultured for the same period of time. The data shown are the means of three independent experiments, each containing at least 12 animals. *Significantly different from the scrambled siRNA control at P<0.05.

A previously described behavioral assay [25], [31] was used to evaluate the effect of cholinergic compounds on *S. mansoni* larval motility. Animals were treated with either cholinergic agonists (arecoline, nicotine) or antagonists (mecamylamine, D-tubocurarine) alone at a concentration of 100 µM and the frequency of body movements (shortening and elongation) was calculated as a measure of motility [25], [31]. Treatment of 6-day old schistosomula with cholinergic agonists caused rapid, near complete paralysis when compared to the water-treated controls (Figure 3A). Conversely, the nicotinic antagonists caused a 2-3.5-fold increase in larval motility. These results are consistent with previous studies [reviewed in 49] and support the hypothesis that cholinergic receptors inhibit neuromuscular function in *S. mansoni*.

To examine the role of the predicted anion-selective nAChR subunits in larval motor behavior, we targeted individual nAChR subunits by RNA interference (RNAi), using pooled sequence–specific siRNAs. A mock–transfected sample (lipid transfection reagent only) and a nonsense scrambled siRNA control were included as negative controls; there was no significant decrease in motor behavior in either control compared to untransfected larvae. In contrast, animals treated with nAChR siRNAs all showed a significant (P<0.05) hyperactive motor phenotype (Figure 3B). Depending on the subunit, the increase in larval motility ranged from 2-4-fold when compared to the negative scrambled control. The two subunits generating very strong hyperactive phenotypes were SmACC-2 (∼6-fold) and SmACC-1 (∼4.5-fold). The hyperactivity in the nAChR RNAi-treated animals is consistent with the phenotype seen in animals where nAChR activity has been pharmacologically abrogated by receptor antagonists (Figure 3A).

**Figure 4:**
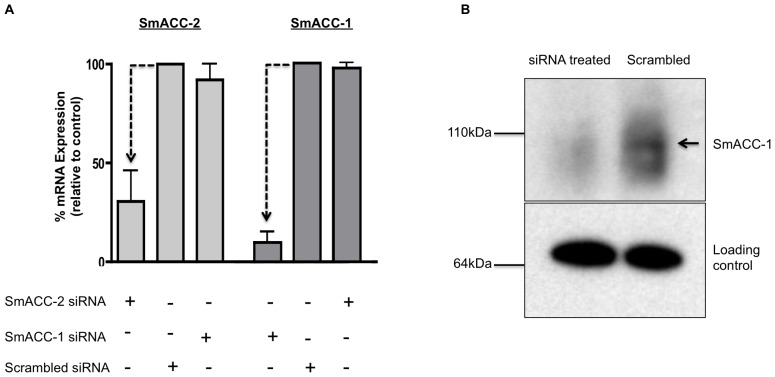
Confirmation of SmACC knockdown. (A) Knockdown of SmACC-1 and SmACC-2 was confirmed at the mRNA level. Schistosomula were transfected with subunit-specific siRNA or scrambled siRNA control, as indicated. RNA was extracted at 6 days post-transfection, oligo-dT reverse-transcribed and quantitative qPCR was performed using primers targeting either a specific subunit or a non-relevant SmACC subunit as an off-target control. Relative expression was calculated using the comparative ΔΔCt method after normalization to a housekeeping gene (GAPDH). The data are derived from three independent experiments, each with 3 replicates, and are shown as the % remaining expression relative to the scrambled siRNA control. Silencing of both subunits was statistically significant, as measured by a Student's t-test at P<0.05 (B) Western blot analysis was performed to assay for silencing of SmACC-1 at the protein level. Crude membrane protein extracts from SmACC-1 siRNA-treated and negative control schistosomula were resolved on a SDS-PAGE gel, transferred to a PVDF membrane and probed with affinity-purified anti-SmACC-1 or a loading control (anti-Sm5-HTR [74]). A band of the expected size (arrow) is present in the scrambled negative control lane but not in the SmACC-1 siRNA-treated lane.

Knockdown at the mRNA level was confirmed by quantitative qPCR for SmACC-1 and SmACC-2 (Figure 4A). SmACC-2 expression was reduced 60% at the transcript level and SmACC-1 expression was reduced by 90%. In both cases the knockdown was observed only in RNAi-suppressed larvae, indicating the effect was specific. Transfection with SmACC-1 siRNAs had no effect on the expression level of the other subunit, SmACC-2, or vice-versa (Figure 4A). Knockdown at the protein level was confirmed by western blot analysis of SmACC-1, using a specific antibody (Figure 4B). The siRNA-treated animals show a drastic reduction in protein expression, as evidenced by the absence of the expected 92 kDa band in the treated sample lane, whereas no difference was seen in the loading control.

### Immunolocalization of SmACC-1 and SmACC-2

In order to determine the tissue localization of SmACC-1 and SmACC-2, we obtained custom commercial antibodies against each target. Polyclonal antibodies were generated using two unique peptide antigens for each gene of interest, each peptide being conjugated to ovalbumin. The antibodies were peptide affinity-purified and tested by ELISA and western blotting. Adult worm membrane fractions probed with anti-SmACC-1 antibody showed a predominant band at ≈100 kDa. Probing with antibodies specific for SmACC-2 resulted in a single band of ≈ 65 kDa. These bands are slightly larger than the predicted sizes (92 kDa and 60 kDa, respectively), possibly due to glycosylation of the native proteins. Control samples in which the antibody was pretreated with an excess of peptide antigen (preadsorbed control) showed no immunoreactivity, indicating specificity of binding for the intended protein.

**Figure 5:**
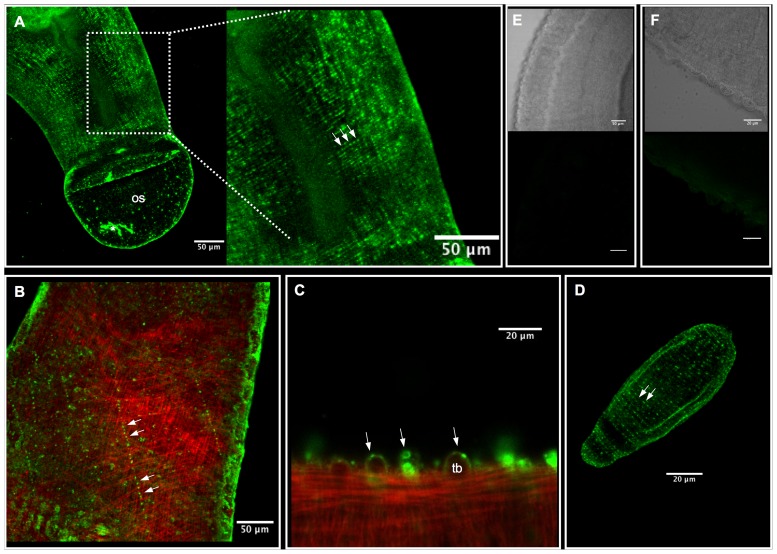
Immunolocalization of SmACC-1 and SmACC-2 in *Schistosoma mansoni*. Adult and 6-day old schistosomula were fixed and incubated with affinity-purified anti-SmACC-1 or anti-SmACC-2, followed by Alexa 488-conjugated secondary antibody (green). In some animals the body wall musculature was counterstained with tetramethylrhodamine B isothiocyanate (TRITC)-labeled phalloidin (red). (A) A Z-projection of SmACC-1 immunoreactivity in an adult male worm. SmACC-1 is present in both the oral sucker (os) and in minor nerve fibers of the peripheral innervation of the worm's body wall. The nerve fibers are varicose in appearance, resembling beads on a string (enlarged region, solid arrows) and are repeated along the length of the body. The asterisk (*) indicates an area of non-specific fluorescence resulting from tissue damage (B) Z-projection of an adult male worm labeled with anti-SmACC-2 (green) and phalloidin (red). SmACC-2 immunoreactivity is present in varicose nerve fibers (solid arrows) that cross the body in a mesh-like pattern indicative of PNS staining. SmACC-2 and the phalloidin –stained body wall musculature are present at different depths of the animal, suggesting that SmACC-2 does not directly innervate muscle. (C) Tubercles (tb) of an adult male worm labeled with anti-SmACC-2 and phalloidin. Specific, punctate SmACC-2 immunoreactivity can be seen along the surface and within the tubercles (arrows). (D) SmACC-2 forms a pattern of concentric, varicose nerve fibers that run the entire length of a 6-day old schistosomulum. A similar expression pattern was observed in schistosomula labeled with anti-SmACC-1 antibody (not shown). (E) Transmitted light and corresponding fluorescent image of a negative control worm labeled with peptide-preadsorbed anti-SmACC-1 and (F) the same negative control for peptide-preadsorbed anti-SmACC-2. The scale bars for the two negative controls are 50 µm (panel E) and 20 µm (panel F).

For the immunolocalization study, adult and larval schistosomes were stained with either anti-SmACC-1 or anti-SmACC-2 and an Alexa-488 conjugated secondary antibody. Some animals were counterstained with TRITC-conjugated phalloidin to label muscle and cytoskeletal features. The results suggest that SmACC-1 and SmACC-2 are both localized to the peripheral nervous system (PNS) of the worm (Figure 5), a region that is rich in cholinergic neurons [50], [51]. Cholinergic neurons are also present in the brain and main nerve cords of the central nervous system (CNS) [50], [51] but we did not observe significant labeling in these regions, either with anti-SmACC-1 or anti-SmACC-2 antibodies. Within the PNS, SmACC-1 immunoreactivity can be seen in fine varicose nerve fibers, resembling beads on a string, which are repeated along the length of the body (Figure 5 A). Close inspection of the confocal stacks suggests these are minor nerve cords of the vast submuscular plexus that innervates the body wall muscles [52]. Similarly anti-SmACC-2 staining revealed numerous varicose nerve fibers in the peripheral innervation of the body wall (Figure 5B). Some of these nerve fibers can be seen criss-crossing the length of the body, where they come into close contact with the musculature. However there was no visible overlay between the antibody labeling (green) and the phalloidin-stained muscles (red), either for SmACC-2 (Figure 5B) or SmACC-1, suggesting these receptors are expressed in nerve tissue rather than the muscle itself. Other regions where specific immunoreactivity was detected included the nerve plexuses of the suckers, which were labeled by both anti-SmACC-1 and 2 antibodies, and the surface of the worm. Surface labeling was observed only with the anti-SmACC-2 antibody and it occurred in both males and females, though it was particularly enriched in the male tubercles (Figure 5C). It is unknown if this labeling is associated with the tegument itself or possibly sensory nerve endings that are present on the surface of the worm. No comparable fluorescence could be seen in any of the negative controls tested, including a peptide-preadsorbed antibody control (Figure 5E, F) and therefore the labeling is considered to be specific.

Immunolocalization studies were repeated in larval schistosomula and the labeling patterns of SmACC-1 and 2 were found to be similar. In both cases, immunoreactivity occurred in a network of fine varicose nerve fibers that run just below the surface and along the entire length of the body (Figure 5D). This resembles the expression pattern seen in the adults and suggests the receptor is expressed in the developing PNS of the larvae. As with the adults, we were unable to detect specific labeling in the CNS of the larvae with either antibody.

### SmACC-1 Forms a Functional, Nicotinic Chloride Channel

**Figure 6:**
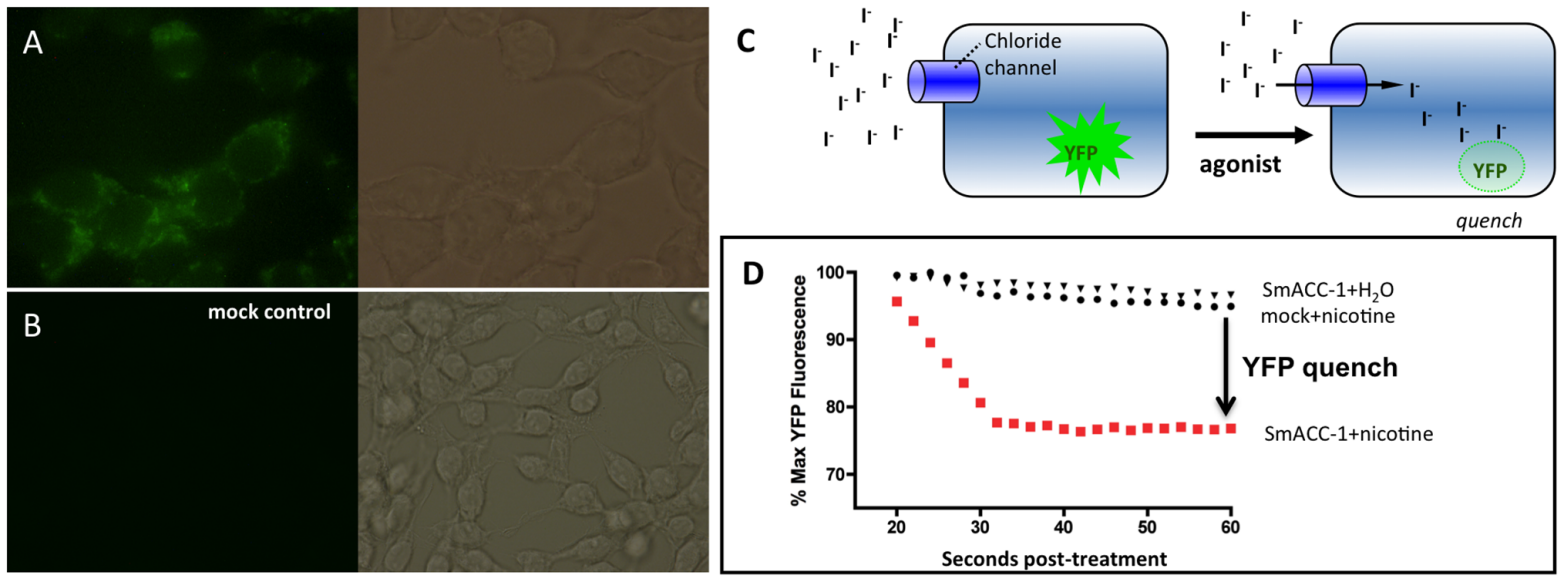
Functional characterization of SmACC-1 in HEK-293 cells. HEK-293 cells were transfected with a human codon-optimized SmACC-1 construct and labeled with affinity-purified anti-SmACC-1 antibody, followed by FITC-conjugated secondary antibody (green). (A) The results show specific immunoreactivity along the surface of the cells, consistent with protein expression. (B) No immunofluorescence is present in cells transfected with empty vector (mock control). (C) Schematic representation of the Premo Halide Sensor YFP quench assay. Cells expressing YFP and the chloride channel of interest are bathed in buffer containing iodide (I^−^), which is used as a surrogate for chloride ions. Agonist-induced activation of the channel causes an influx of I^−^ into the cell and quenches YFP fluorescence. (D) Representative data from individual wells containing cells transfected with either SmACC-1 or empty vector (mock). Treatment of SmACC-1 expressing cells with 100 µM nicotine (solid red squares) resulted in a significant reduction in YFP fluorescence (YFP quench) when compared to both a water-treated negative control (solid triangles) and mock-transfected cells treated with 100 µM nicotine (solid circles). Data were normalized relative to maximum YFP fluorescence for each sample.

HEK-293 cells were transfected with codon-optimized (humanized) SmACC-1 and protein expression was monitored by *in situ* immunofluorescence. SmACC-1 was selected for these studies because it is a predicted alpha-like subunit and therefore it is capable, in principle, of forming functional homomeric channels [10]. Initial attempts to express the native (non-humanized) SmACC-1 proved unsuccessful. The codon-optimized sequence, however, expressed significant levels of protein in the HEK-293 cells. The transfected cells were immunoreactive for SmACC-1 when probed either with specific antibody (Figure 6A) or anti-FLAG antibody targeting the C-terminal FLAG epitope. No immunofluorescence was noted in the negative control cells transfected with empty plasmid (Figure 6B).

Cells expressing codon-optimized SmACC-1 were transduced with a YFP sensor (Premo Halide Sensor) and seeded on a 96-well plate for the iodide (I^−^) flux assay. The principle of the assay has been described in detail [37]–[40] and is shown schematically in Figure 6C. Cells expressing a chloride channel of interest are bathed in an iodide buffer, which serves as a surrogate for chloride (Cl^−^) anions. After a period of equilibration, test compounds are added and if the chloride channel of interest is activated, an influx of I^−^ occurs, quenching the fluorescence of the YFP sensor. Channel activity was quantified by measuring either the slope of the curve or the decrease in fluorescence following drug addition, as described [39]. Figure 6D shows representative tracings of cells expressing SmACC-1 and mock-transfected cells, each treated with 100 µM nicotine. Activation of SmACC-1 (red circles) by nicotine caused a significant decrease in YFP fluorescence compared to nicotine-treated mock-transfected cells (black circles). No significant reduction in fluorescence was seen in SmACC-1 expressing cells treated with water, suggesting the YFP quench was agonist-dependent. In separate experiments, we also tested whether SmACC-1 was able to transport calcium in the HEK-293 cells, using a kit-based calcium fluorescence assay. This was done in part to verify the ion selectivity of the channel and also to address the possibility that the YFP quench might be due to indirect activation of an endogenous calcium-sensitive chloride channel. However these experiments showed no evidence of calcium influx through SmACC-1. Cells expressing SmACC-1 were treated with 100 µM nicotine or 100 µM ACh and there was no effect of either agonist on intracellular calcium levels (data not shown). Thus we rule out an indirect effect of calcium on I^−^ transport and conclude that SmACC-1 is a cholinergic anion channel, as predicted from the bioinformatics analysis.

**Figure 7:**
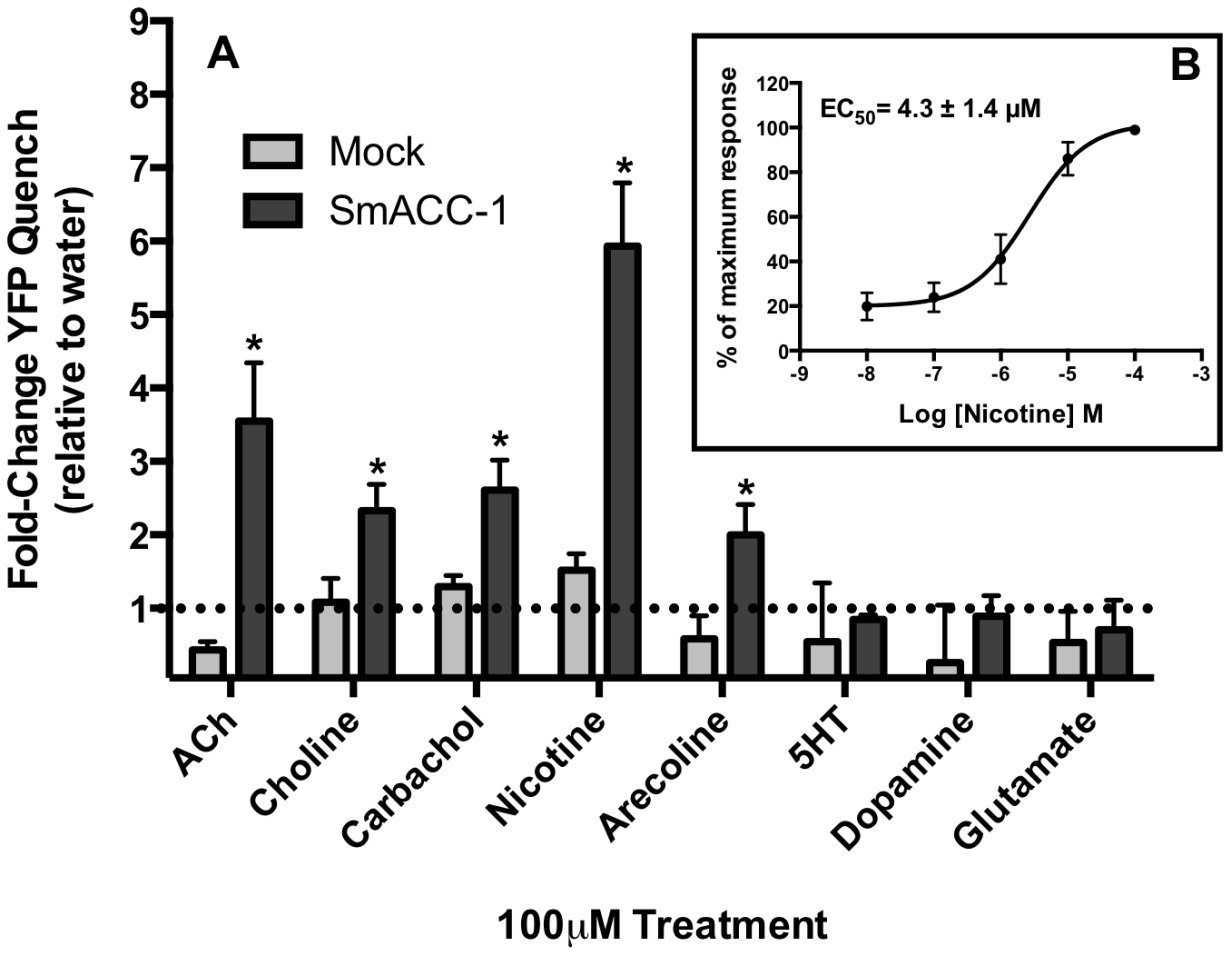
SmACC-1 is selectively activated by cholinergic substances in transfected HEK-293 cells. (A) A panel of cholinergic receptor agonists (acetylcholine (ACh), choline, carbachol, nicotine, arecoline) was tested against SmACC-1 expressing or mock-transfected cells. The YFP quench data were normalized relative to the water-treated control measured in the same experiment and on the same plate. Results are the means and SEM of 3-4 experiments, each containing 6 technical replicates per treatment. All cholinergic agonists caused a significant reduction in YFP fluorescence at P<0.05 (*) compared to the water control. Treatment of SmACC-1-expressing cells with serotonin (5HT), glutamate or dopamine did not result in significant YFP quench. (B) SmACC-1 expressing cells were treated with variable concentrations of nicotine and YFP quench was calculated. The YFP quench data were normalized relative to the maximum response for each experiment and an EC_50_ value was calculated by nonlinear regression analysis of the normalized data. The results are the means ± SEM of 3 independent experiments, each with six replicates.

**Figure 8:**
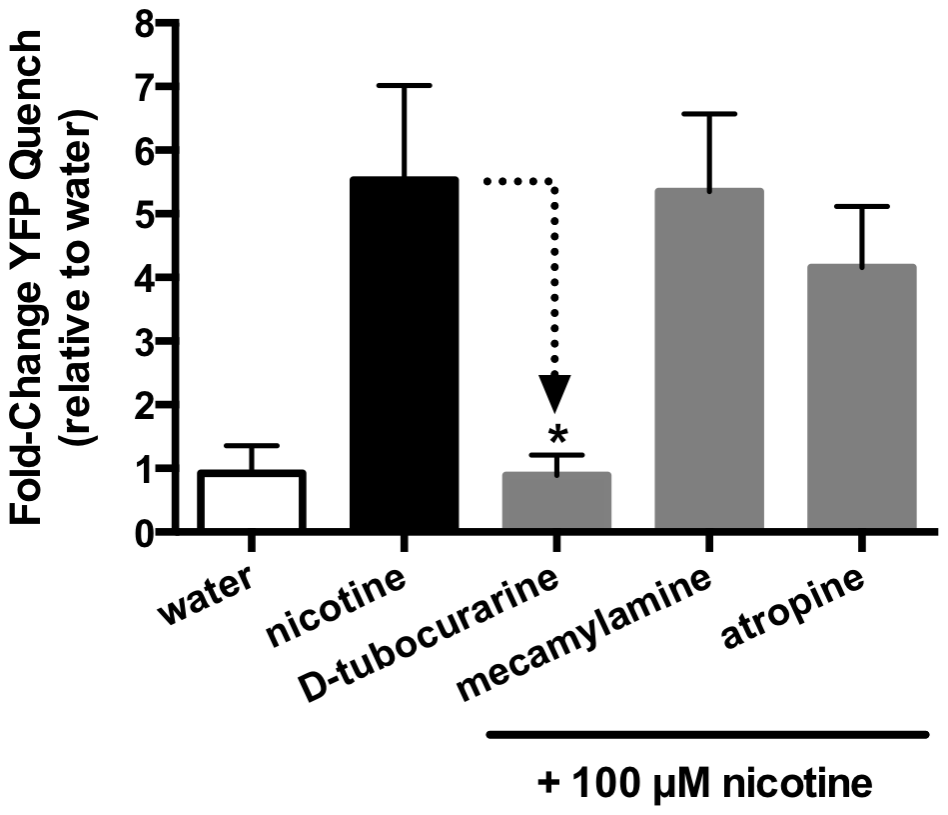
SmACC-1 is selectively antagonized by D-tubocurarine in transfected HEK-293 cells. SmACC-1-expressing cells were pre-incubated with cholinergic antagonists (mecamylamine, D-tubocurarine, atropine), each at a concentration of 100 µM. Cells were then treated with 100 µM nicotine and YFP quench was measured. Control SmACC-1 cells were treated with nicotine in the absence of antagonist. The YFP quench data were normalized relative to the water-treated control. Results are the means and SEM of 12 replicates from two separate transfections.

The I^−^ flux (YFP sensor) experiments were repeated with different test substances and the results are shown in Figure 7. None of the compounds used stimulated a significant influx of I^−^ in the mock control. In contrast the cells expressing SmACC-1 were responsive to several cholinergic agonists, particularly nicotine. Treatment with nicotine (100 µM) caused a significant (P<0.05) ≈6-fold increase in YFP quench in cells expressing SmACC-1. Smaller but statistically significant responses were also seen with other cholinergic agonists (ACh, choline chloride, carbachol and arecoline). Non-cholinergic substances, including biogenic amines (serotonin (5HT), dopamine) and glutamate, had no effect on the cells (Figure 7). These data suggest that SmACC-1 is capable of forming a functional homomeric chloride channel that displays a preference for nicotine and related cholinergic substances. Furthermore, SmACC-1 was activated by nicotine in a dose-dependent manner with an EC_50_  =  4.3±1.4 µM (Figure 7, inset). To test if the channel is sensitive to inhibition by cholinergic antagonists, SmACC-1 – expressing cells were treated with nicotine (100 µM) in the presence and absence of “classical” (mammalian) nicotinic antagonists (D-tubocurarine, mecamylamine) or the muscarinic (GAR) antagonist, atropine, each at 100 µM. Of the drugs tested, only D-tubocurarine was able to significantly block the activation of SmACC-1 by nicotine (Figure 8). The other two drugs, mecamylamine and atropine were ineffective at this concentration.

## Discussion

Acetylcholine (ACh) has long been known as the quintessential excitatory neurotransmitter of the vertebrate neuromuscular system. Signaling through cation-selective nAChRs, ACh mediates muscular contraction via membrane depolarization due to an influx of Na^+^ or Ca^2+^. More recently, a distinct class of anion-selective nAChRs and other types of acetylcholine-gated chloride channels (ACCs) has been reported in several invertebrate organisms, including mollusks and nematodes [11], [12]. These chloride-permeable channels initiate membrane hyperpolarization, causing an inhibition of action potentials. However, none of these invertebrate channels has been directly implicated in the control of motor function.

The effects of ACh on invertebrate neuromuscular activity vary depending upon the organism in question. As in vertebrates, ACh has excitatory neuromuscular effects in many invertebrate phyla, including some helminths such as nematodes and planarians [53], [54]. In trematodes, however, ACh appears to act in exactly the opposite manner. Exogenous application of cholinergic agonists onto trematodes in culture causes a rapid flaccid paralysis due to relaxation of the body wall muscles [15], [55]. A similar type of paralysis was observed in tapeworms (cestodes) treated with exogenous ACh [16]. This inhibitory response to cholinergic drugs appears unique to parasitic flatworms (trematodes and cestodes), and the receptors mediating this activity may therefore hold value as a therapeutic target. Earlier electrophysiology studies of *S. mansoni* tentatively identified these receptors as nAChR-like based on their pharmacological properties [17] but the receptors themselves were not identified. The sequencing of the *S. mansoni* genome [18]–[19] led to the annotation of several candidate nAChR subunit genes, which are the focus of the present work.

Using a combination of BLAST and keyword searches, a total of nine nAChR subunit genes were found in the genome of *S. mansoni*. A structural alignment of the schistosome nAChR subunits with the *Torpedo* nAChR was then performed to identify peptide motifs associated with ion-selectivity. Cation-selective ion channel subunits have a negatively charged intermediate ring, formed by the presence of Glu residues in the M1-M2 linking region [56]. Anion-selective Cys-loop receptor subunits replace the Glu in this region with a Pro-Ala motif, disrupting the electrostatic interactions in the intermediate ring and conferring anion-selectivity to the channel [14, 45, 46 see 47 for review]. The results of our structural alignment indicate that 5 of the schistosome nAChR subunits (SmACC-1, SmACC-2, Smp_157790, Smp_037910 and Smp_132070) contain this anion-selectivity determinant and they were tentatively identified as *S. mansoni* SmACCs. Furthermore, a dendrogram analysis suggests that the SmACCs are evolutionarily distinct from the ACCs found in *C. elegans*. Unlike the *C. elegans* ACCs [12], the schistosome subunits are structurally related to vertebrate and invertebrate nAChRs, suggesting that the SmACCs are descended from ancient nicotinic channels but have evolved selectivity for chloride. This allies the SmACCs more closely with the anion-selective nAChRs of the snail *Lymnaea* [11], with which they share more than 40% identity at the protein level. Interestingly, certain species of *Lymnaea* are permissive intermediate hosts of schistosomes. However, it is unclear if the presence of anion-selective nicotinic channels in both organisms is due to horizontal gene transfer, common ancestry or convergent evolution. There is also evidence of closely related, putative nAChR chloride channels present in the genome of the trematode *Clonorchis sinensis* [57], suggesting a unique clade of platyhelminth-specific nicotinic chloride channels.

The next step after identifying the SmACCs was to study their role in the motor function of the parasite. The flaccid paralysis of adult schistosomes caused by treatment with cholinergic compounds is well characterized. However, very little is known about the role of cholinergic receptors in the motor activity of larval schistosomula. Given that larval migration is vital to parasite development and survival [6] and the cholinergic system is a major regulator of motor function in adult worms, we hypothesized that SmACCs play an important role as inhibitory modulators in larval neuromuscular function. To test this, two types of behavioral assay were employed- pharmacological and RNAi. The results of the pharmacological motility assay agree with previous studies implicating ACh as an inhibitor of schistosome movement [15], [17]. Treatment of 6-day old schistosomula with the cholinergic agonists arecoline and nicotine caused nearly complete paralysis whereas classical antagonists, mecamylamine and D-tubocurarine stimulated movement by 3–4 fold over water-treated control animals. These results suggest that the schistosome cholinergic system mediates inhibitory neuromuscular responses, possibly via an influx of chloride generated by SmACC activation.

Although the results of the pharmacological motility assay agree with previously published studies, motor phenotypes elicited by treatment of worms with exogenous compounds are not necessarily of biological or behavioral relevance. Drug permeability across the tegument, non-selective targeting and toxic effects may all induce motor behaviors that obscure the role of the receptors in question. Silencing of receptor function by RNAi mitigates these issues by targeting receptors individually and by measuring effects on basal motor activity in the absence of added drugs. The results of our RNAi assay show that the ion channels formed by the SmACC subunits act as inhibitory mediators of motor activity in schistosomula. Knockdown of each of the 5 identified SmACC subunits resulted in a 3-6-fold hypermotile phenotype, mirroring the hyperactivity seen in antagonist-treated schistosomula. It is unclear why the individual subunits all produced similar hypermotile RNAi phenotypes. It is possible these are all components of the same inhibitory channel, such that the loss of any one subunit results in loss of channel function and hyperactivity. As discussed below, our immunolocalization studies show that two of these subunits, at least (SmACC-1 and SmACC-2) have similar distribution patterns, suggesting they could be components of the same channel in the worm. Alternatively these could assemble into different channels that have similar inhibitory effects on movement.

To identify the possible mechanisms by which the SmACCs mediate inhibitory motor responses, immunolocalization studies were performed by confocal microscopy. The tissue distribution of two SmACCs in which silencing elicited large hypermotile phenotypes, SmACC-1 and SmACC-2, was examined in adult and larval stages of the parasite. The most significant expression was observed in the peripheral innervation of the worm's body wall, both for SmACC-1 and SmACC-2. Counterstaining with phalloidin suggests that neither subunit is expressed directly on the musculature. Rather, SmACC-1 and SmACC-2 were detected in minor nerve fibers of the submuscular nerve net that innervates the somatic muscles. This suggests that SmACC-1 and SmACC-2 mediate their inhibitory motor effects in an indirect manner, perhaps by modulating the release of other neurotransmitters or by acting as autoreceptors. In flatworms, as well as vertebrate model systems, nicotinic receptors are well known to mediate the release of other neurotransmitters, including neuropeptides and dopamine [58]–[60]. In schistosomes, the cholinergic and neuropeptidergic system (which is excitatory in flatworms), are in very close proximity [50], [61]. The balance between these systems may, therefore, be an important factor in the regulation of motor behavior. It would be of interest to determine if ACh inhibits neuropeptide release through these receptors, and whether this inhibition might explain the flaccid paralysis and other motor effects of ACh in these parasites.

SmACC-2 immunoreactivity was also seen on the surface of the parasite. Discreet, punctate staining is present along and in between the tubercles of adult male worms and along the surface of adult females. This marks the second time a nAChR has been localized to the schistosome tegument [62]. Surface nAChRs in schistosomes have previously been linked to modulation of glucose uptake and are postulated to act through tegumental GLUT-1 like transporters [63]. The possibility also exists that tegumental SmACC-2 may provide sensory cues affecting motor function. The tubercles are known to contain innervated sensory structures [64], which interface with the peripheral nerve net below and ultimately the CNS. The presence of SmACC-2 at both of these locations points to a potential role for ACh and this receptor in mediating host-parasite interactions affecting worm motor behavior.

While behavioral assays and microscopy serve to elucidate the behavioral role of the SmACCs, they provide only limited insight into receptor function at the molecular level. Therefore, functional expression analysis of a SmACC receptor was carried out in a heterologous expression system. A previous study cloned and expressed two cation-selective nAChR subunits from *S. haematobium* in *Xenopus* oocytes [65]. However, neither subunit was able to form a functional ion channel either alone or when co-expressed. Our initial attempts to express SmACC-1 and SmACC-2 failed to produce functional channels, either individually or in combination and in two different expression environments, HEK-293 cells and *Xenopus* oocytes (data not shown). SmACC-2 lacks the YxCC motif of nAChR alpha-subunits and therefore is not capable of forming functional homomeric channels. Further examination with appropriate antibodies of cells transfected with the SmACC-1 subunit determined that the level of protein expression was low, which could explain the apparent lack of activity. It has been shown that differences in codon-usage can significantly decrease the expression of recombinant schistosome proteins in heterologous systems [66]. Thus we obtained a codon-optimized (humanized) cDNA for SmACC-1 and repeated the analysis in HEK-293 cells. The humanized construct produced higher levels of protein expression and some of this protein appeared to be correctly targeted to the cell surface, as determined by immunofluorescence analysis. Subsequent functional studies showed that human codon-optimized SmACC-1 produced a functional homomeric ion channel in HEK-293 cells. Several nAChR subunits are known to form functional homomeric channels *in vivo*. Examples of this include the vertebrate alpha-7 nAChR and the ACR-16 of *C. elegans* [67]–[68]. However, the expression of functional homomeric nAChRs is limited to neuronally expressed channels [69]. Moreover, only alpha-type nAChR subunits are capable of forming homopentameric channels. Thus, the formation of a functional homomeric SmACC-1 channel, together with its neuronal expression pattern in the worm, both suggest that SmACC-1 is a neuronal-type alpha nAChR subunit.

Activity assays were performed using a relatively novel, fluorescence-based assay, the Premo Halide Sensor (Invitrogen). The results of the activity assay show that SmACC-1 is activated by cholinergic agonists but not other biogenic amines. Nicotine and ACh induced the largest response (≈ 6-fold and 2.5-fold, respectively) when compared to water-treated control cells. An EC_50_ of 4.3 µM was calculated for nicotine, which falls within the reported range for vertebrate neuronal nAChR response to nicotine, as well as an nAChR characterized in the parasitic nematode *A. suum* [70]–[72]. Subsequent pharmacological studies showed that the response to nicotine was virtually abolished by D-tubocurarine, suggesting the drug effects on movement are mediated, at least in part, by this subunit. In contrast, mecamylamine had no effect on the recombinant channel and therefore it must be acting through nAChRs that do not involve SmACC-1. Interestingly, the closely related *Lymnae* ACh-gated chloride channel was also reported to be insensitive to mecamylamine [11].

Functional analysis of SmACC-1 in a mammalian expression system represents a departure from the more classical electrophysiological method in *Xenopus* oocytes. Although electrophysiological characterization is the gold standard for measurement of ion channel activity, this method is technically demanding, labor-intensive and generally unsuitable for screening large numbers of compounds. In order to mitigate these issues, researchers have turned to mammalian cell-based ion channel functional assays. Expression of target ion channels in mammalian cells still allows direct measurement of ion flux and membrane potential, however it does so in a high-throughput format. Assays exist for a variety of ion channel types (Ca^2+^, Na^+^, Cl-) and many are commercially available [reviewed in 73]. Moreover, the data from these HTS assays generally correlate well with results generated by traditional electrophysiological methods [73]. The Premo Halide Assay employed in this study is based upon technology used to identify small molecule inhibitors of CFTR chloride channels [37]. The high-throughput format of the assay allows for the possibility of screening large chemical libraries against parasite receptors that may have highly divergent pharmacology. Given the major effects the SmACCs exert over worm motor function, this is an option worth pursuing.

The work described here adds to the mounting evidence of acetylcholine's role as a major inhibitory transmitter in schistosomes. We have described a novel clade of nicotinic acetylcholine-gated chloride channel subunits (SmACCs) that are phylogenetically distant from the *C. elegans* ACCs and play a major role in inhibitory neuromuscular modulation as it pertains to larval motor behavior. The localization of the SmACCs to the peripheral nervous system points to their broad, indirect role in this modulation. Functional studies in mammalian cells indicate that the SmACC subunits are capable of forming functional nicotinic chloride channels *in vitro*. Finally, the use of a fluorescent, mammalian cell-based functional assay for a helminth ion channel represents a new tool in the search for new anti-schistosomal drugs.

## Supporting Information

Figure S1 **Validation of anti-SmACC antibodies in adult schistosomes.** Crude membrane protein extract from adult *S. mansoni* was run on an SDS-PAGE gel, transferred to a PVDF membrane and probed with affinity-purified anti-SmACC-1 antibody (A) or anti-SmACC-2 antibody (B), followed by horseradish peroxidase (HRP) conjugated secondary antibody. The positions of the two immunoreactive bands are indicated. There was no immunoreactivity in the antigen-preadsorbed negative control for either antibody.
(TIF)

Table S1 **List of Cys-loop receptor sequences used for phylogenetic analysis of SmACCs.**
(XIS)

Table S2 **List of PCR primers used for generation of siRNA and qPCR of SmACCs.**
(XIS)

## References

[1] Gryseels B, Polman K, Clerinx J, Kestens L (2006) Human Schistosomiasis. Lancet 368(9541): 1106–1118. 16997665 10.1016/S0140-6736(06)69440-3

[2] Doenhoff MJ, Hagan P, Cioli D, Southgate V, Pica-Mattoccia L, et al (2009) Praziquantel: its use in control of schistosomiasis in sub-Saharan Africa and current research needs. Parasitology 136(13): 1825–35. 19281637 10.1017/S0031182009000493

[3] Melman SD, Steinauer ML, Cunningham C, Kubatko LS, Mwangi IN, et al (2009) Reduced Susceptibility to Praziquantel among Naturally Occurring Kenyan Isolates of *Schistosoma mansoni* . PLoS Negl Trop Dis 3(8): e504. 19688043 10.1371/journal.pntd.0000504 PMC2721635

[4] Sabah AA, Fletcher C, Webbe G, Doenhoff J (1986) *Schistosoma mansoni*: chemotherapy of infections of different ages. Exp Parasitol 61: 294–303. 3086114 10.1016/0014-4894(86)90184-0

[5] Robertson AP, Martin RJ (2007) Ion-channels on parasite muscle: pharmacology and physiology. Invert Neurosci 7(4): 209–17. 17999098 10.1007/s10158-007-0059-x

[6] Crabtree JE, Wilson RA (1980) *Schistosoma mansoni*: a scanning electron microscope study of the developing schistosomulum. Parasitology 81(Pt 3): 553–64. 7232034 10.1017/s003118200006193x

[7] Maule AG, Day TA, Chappell CH (2005) Parasite neuromusculature and its utility as a drug target. Parasitology 131: S1–S2.

[8] Kaminsky R, Gauvry N, Schorderet Weber S, Skripsky T, Bouvier J, et al (2008) Identification of the amino-acetonitrile derivative monepantel (AAD 1566) as a new anthelminthic drug development candidate. Parasitol Res 103(4): 931–939. 18594861 10.1007/s00436-008-1080-7 PMC2491438

[9] Bueding E, Liu CL, Rogers SH (1972) Inhibition by metrifonate and dichlorvos of cholinesterases in schistosomes. Br J Pharmacol 46(3): 480–7. 4656609 10.1111/j.1476-5381.1972.tb08145.x PMC1666567

[10] Albuquerque EX, Pereira EF, Alkondon M, Rogers SW (2009) Mammalian nicotinic acetylcholine receptors: from structure to function. Physiol Res 89(1): 73–120. 10.1152/physrev.00015.2008 PMC2713585 19126755

[11] van Nierop P, Keramidas A, Bertrand S, van Minnen J, Gouwenberg Y, et al (2005) Identification of Molluscan Nicotinic Acetylcholine Receptor (nAChR) Subunits Involved in Formation of Cation- and Anion-Selective nAChRs. J Neurosci 25(46): 10617–10626. 16291934 10.1523/JNEUROSCI.2015-05.2005 PMC6725845

[12] Putrenko I, Zakikhani M, Dent JA (2005) A Family of Acetylcholine-gated Chloride Channel Subunits in *Caenorhabditis elegans* . J Biol Chem 280: 6392–6398. 15579462 10.1074/jbc.M412644200

[13] Beech RN, Callanan MK, Rao VTS, Dawe GB, Forrester SG (2013) Characterization of Cys-loop receptor genes involved in inhibitory amine neurotransmission in parasitic and free-living nematodes. Parasitology Int 62: 599–605. 10.1016/j.parint.2013.03.010 23602737

[14] Keramidas A, Moorhouse AJ, Pierce KD, Schofield PR, Barry PH (2002) Cation-selective Mutations in the M2 Domain of the Inhibitory Glycine Receptor Channel Reveal Determinants of Ion-Charge Selectivity. J Gen Physiol 119: 393–410. 11981020 10.1085/jgp.20028552 PMC2233820

[15] Barker LR, Bueding E, Timms AR (1966) The possible role of acetylcholine in *Schistosoma mansoni* . Brit J Pharmacol 26: 656–665. 10.1111/j.1476-5381.1966.tb01845.x PMC1510701 4381202

[16] Wilson CVLC, Schiller EL (1969) The neuroanatomy of *Hymenolepis dimimuta* and *H. nana* . J Parasitol 55(2): 261–70. 4305582

[17] Day TA, Chen GZ, Miller C, Tian M, Bennett JL, Pax RA (1996) Cholinergic inhibition of muscle fibers isolated from *Schistosoma mansoni* (Trematoda: Digenea). Parasitology 113 (Pt. 1): 55–61. 10.1017/s0031182000066270 8710415

[18] Berriman M, Haas BJ, LoVerde PT, Wilson RA, Dillon GP, et al (2009) The genome of the blood fluke *Schistosoma mansoni* . Nature 460: 352–358. 19606141 10.1038/nature08160 PMC2756445

[19] Protasio AV, Tsai IJ, Babbage A, Nichol S, Hunt M (2012) A systematically improved high quality genome and transcriptome of the human blood fluke *Schistosoma mansoni* . PLoS Negl Trop Dis 6(1): e1455. 22253936 10.1371/journal.pntd.0001455 PMC3254664

[20] Behm CA, Bendig MM, McCarter JP, Sluder AE (2005) RNAi-based discovery and validation of new drug targets in filarial nematodes. Trends Parasitol 21(3): 97–100. 15734653 10.1016/j.pt.2004.12.003

[21] Boyle JP, Wu XJ, Shoemaker CB, Yoshino TP (2003) Using RNA interference to manipulate endogenous gene expression in *Schistosoma mansoni* sporocysts. Mol Biochem Parasitol 128(2): 205–15. 12742587 10.1016/s0166-6851(03)00078-1

[22] Kreutz-Peterson G, Radwanska M, Ndegua D, Shoemaker CK, Skelly PJ (2007) Optimizing gene suppression in schistosomes using RNA interference. Mol Biochem Parasitol 153(2): 194–202. 17420062 10.1016/j.molbiopara.2007.03.006

[23] Reddien PW, Bermange AL, Murfitt KJ, Jennings JR, Sánchez Alvarado A (2005) Identification of genes needed for regeneration, stem cell function, and tissue homeostasis by systematic gene perturbation in planaria. Dev Cell 8(5): 635–49. 15866156 10.1016/j.devcel.2005.02.014 PMC2267917

[24] McVeigh P, Mair GR, Novozhilova E, Day A, Zamanian M, Marks NJ, Kimber MJ, Day TA, Maule AG (2011) Schistosome I/Lamides—a new family of bioactive helminth neuropeptides. Int J Parasitol 41(8): 905–13. 21554884 10.1016/j.ijpara.2011.03.010 PMC3118037

[25] Patocka N, Ribeiro P (2013) The functional role of a serotonin transporter in *Schistosoma mansoni* elucidated through immunolocalization and RNA interference (RNAi). Mol Biochem Parasitol 187: 32–42. 23246818 10.1016/j.molbiopara.2012.11.008

[26] Lewis F (2001) Schistosomiasis. Current Protocols in Immunology 28: 19.1.1–19.1.28. 10.1002/0471142735.im1901s28 PMC4034062 18432750

[27] Larkin MA, Blackshields G, Brown NP, Chenna R, McGettigan PA, et al (2007) Clustal W and Clustal X version 2.0. Bioinformatics 23(21): 2947–2948. 17846036 10.1093/bioinformatics/btm404

[28] Felsenstein J (1989) PHYLIP - Phylogeny Inference Package (Version 3.2). Cladistics 5: 164–166.

[29] Morariu VI, Srinivasan BV, Raykar VC, Duraiswami R, Davis LS (2008) Automatic online tuning for fast Gaussian summation. Advances in Neural Information Processing Systems (NIPS) 2007. Available: http://books.nips.cc/papers/files/nips21/NIPS2008_0257.pdf.

[30] Rychlik W (2007) OLIGO 7 Primer Analysis Software. In: Yuryev A, editor. Methods in Molecular Biology Vol. 402: PCR Primer Design. Totowa: Humana Press. pp 35–59. 10.1007/978-1-59745-528-2_2 17951789

[31] El-Shehabi F, Taman A, Moali LS, El-Sakkary N, Ribeiro P (2012) A novel G protein-coupled receptor of *Schistosoma mansoni* (SmGPR-3) is activated by dopamine and is widely expressed in the nervous system. PLoS Negl Trop Dis 6(2): e1523. 22389736 10.1371/journal.pntd.0001523 PMC3289605

[32] Gold D (1997) Assessment of the viability of *Schistosoma mansoni* schistosomula by comparative uptake of various vital dyes. Parasitol Res 83: 163–169. 9039699 10.1007/s004360050227

[33] Livak KJ, Schmittgen TD (2001) Analysis of relative gene expression data using real-time quantitative PCR and the 2(-Delta Delta C(t)) Method. Methods 25(4): 402–8. 11846609 10.1006/meth.2001.1262

[34] Mair GR, Maule AG, Day TA, Halton DW (2000) A confocal microscopical study of the musculature of adult *Schistosoma mansoni* . Parasitology 121(Pt 2): 163–170. 11085236 10.1017/s0031182099006174

[35] Taman A, Ribeiro P (2009) Investigation of a dopamine receptor in *Schistosoma mansoni*: Functional studies and immunolocalization. Mol Biochem Parasitol 168: 24–33. 19545592 10.1016/j.molbiopara.2009.06.003

[36] Collins JJ III, King RS, Cogswell A, Williams DL, Newmark PA (2011) An Atlas for *Schistosoma mansoni* Organs and Life-Cycle Stages Using Cell Type-Specific Markers and Confocal Microscopy. PLoS Negl Trop Dis 5(3): e1009. 21408085 10.1371/journal.pntd.0001009 PMC3050934

[37] De La Fuente R, Namkung W, Mills A, Verkman AS (2008) Small-molecule screen identifies inhibitors of a human intestinal calcium-activated chloride channel. Mol Pharmacol 73: 758–768. 18083779 10.1124/mol.107.043208

[38] Galietta LJ, Haggie PM, Verkman AS (2001) Green fluorescent protein-based halide indicators with improved chloride and iodide affinities. FEBS Lett 499: 220–224. 11423120 10.1016/s0014-5793(01)02561-3

[39] Johansson T, Norris T, Peilot-Sjögren H (2013) Yellow Fluorescent Protein-Based Assay to Measure GABA_A_ Channel Activation and Allosteric Modulation in CHO-K1 Cells. PLoS ONE 8(3): e59429. 23516634 10.1371/journal.pone.0059429 PMC3597608

[40] Verkman AS, Galietta LJ (2009) Chloride channels as drug targets. Nat Rev Drug Discov 8(2): 153–71. 19153558 10.1038/nrd2780 PMC3601949

[41] Kruger W, Gilbert D, Hawthorne R, Hryciw DH, Frings S, Poronnik P Lynch JW (2005) A yellow fluorescent protein-based assay for high-throughput screening of glycine and GABAA receptor chloride channels. Neurosci Lett 380(3): 340–5. 15862914 10.1016/j.neulet.2005.01.065

[42] Xie J, Dernovici S, Ribeiro P (2005) Mutagenesis analysis of the serotonin 5-HT2C receptor and a *Caenorhabditis elegans* 5HT2 homologue: Conserved residues of helix 4 and 7 contribute to agonist-dependent activation of 5HT2 receptors. J Neurochem 92: 375–387. 15663485 10.1111/j.1471-4159.2004.02867.x

[43] Nabhan J, Ribeiro P (2006) The 19S proteasomal subunit POH1 contributes to the regulation of c-Jun ubiquitination, stability and subcellular localization. J Biol Chem 281: 16099–16107. 16569633 10.1074/jbc.M512086200

[44] Thompson AJ, Lester HA, Lummis SCR (2010) The Structural Basis of Function in Cys-Loop Receptors. Q Rev Biophys 43(4): 449–99. 20849671 10.1017/S0033583510000168

[45] Galzi J-L, Devillers-Thiery A, Hussy N, Bertrand S, Changeux J-P, Bertrand D (1992) Mutations in the channel domain of a neuronal nicotinic receptor convert ion selectivity from cationic to anionic. Nature 359: 500–505. 1383829 10.1038/359500a0

[46] Corringer P-J, Bertrand S, Galzi J-L, Devillers-Thiery A, Changeux J-P, Bertrand D (1999) Mutational analysis of the charge selectivity filter of the α7 nicotinic acetylcholine receptor. Neuron 22: 831–843. 10230802 10.1016/s0896-6273(00)80741-2

[47] Karlin A (2002) Emerging structure of the nicotinic acetylcholine receptors. Nature Rev (Neurosc) 3: 102–114. 10.1038/nrn731 11836518

[48] Kao PN, Karlin A (1996) Acetylcholine receptor binding site contains a disulfide cross-link between adjacent half-cystinyl residues. J Biol Chem 261(18): 8085–8088. 3722144

[49] Ribeiro P, El-Shehabi F, Patocka N (2005) Classical transmitters and their receptors in flatworms. Parasitology (131): S19–S40. 16569290 10.1017/S0031182005008565

[50] Halton DW, Gustaffson MKS (1996) Functional morphology of the platyhelminth nervous system. Parasitology (113): S47–S72.

[51] Nishimura K, Kitamura Y, Taniguchi T, Agata K (2010) Analysis of motor function modulated by cholinergic neurons in planarian Dugesia japonica. Neurosci 168(1): 18–30. 10.1016/j.neuroscience.2010.03.038 20338223

[52] Reuter M, Gustafsson MKS (1995) The flatworm nervous system: Pattern and phylogeny. *In* The nervous systems of invertebrates: An evolutionary and comparative approach (Breidbach O, Kutsch W, eds) pp. 25–59. 10.1007/978-3-0348-9219-3_3 7833618

[53] Walker RJ, Franks CJ, Pemberton D, Rogers C, Holden-Dye L (2000) Physiological and pharmacological studies on nematodes. Acta Biol Hung 51(2-4): 379–94. 11034163

[54] Butarelli FR, Pontieri FE, Margotta V, Palladini G (2000) Acetylcholine/dopamine interaction in planaria. Comp Biochem Physiol C Toxicol Pharmacol 125(2): 225–31. 11790344 10.1016/s0742-8413(99)00111-5

[55] Holmes FP, Fairweather I (1984) *Fasciola hepatica*: the effects of neuropharmacological agents in *in vitro* motility. Exp Parasitol 58: 194–208. 6148259 10.1016/0014-4894(84)90035-3

[56] Wilson GG, Pascual JM, Brooijmans N, Murray D, Karlin A (2000) The Intrinsic Electrostatic Potential and the Intermediate Ring of Charge in the Acetylcholine Receptor Channel. J Gen Physiol 115: 93–106. 10653890 10.1085/jgp.115.2.93 PMC2217203

[57] Huang Y, Chen W, Wang X, Liu H, Chen Y, et al (2013) The carcinogenic liver fluke, *Clonorchis sinensis*: new assembly, reannotation and analysis of the genome and characterization of tissue transcriptomes. PLOS One 8(1): e54732. 23382950 10.1371/journal.pone.0054732 PMC3559784

[58] Barik J, Wonnacott S (2006) Indirect Modulation by _7 Nicotinic Acetylcholine Receptors of Noradrenaline Release in Rat Hippocampal Slices: Interaction with Glutamate and GABA Systems and Effect of Nicotine Withdrawal. Molec Pharmacol 69(2): 618–628. 16269536 10.1124/mol.105.018184

[59] Akasu T, Ohta Y, Koketsu K (1984) Neuropeptides facilitate the desensitization of nicotinic acetylcholine-receptor in frog skeletal muscle endplate. Brain Res 290(2): 342–347. 6198044 10.1016/0006-8993(84)90953-3

[60] Di Angelantonio S, Giniatullin R, Costa V, Sokolova E, Nistri A (2003) Modulation of neuronal nicotinic receptor function by the neuropeptides CGRP and substance P on autonomic nerve cells. Br J Pharmacol 139(6): 1061–73. 12871824 10.1038/sj.bjp.0705337 PMC1573932

[61] Halton DW, Maule AG (2004) Flatworm nerve–muscle: structural and functional analysis. Can J Zool 82(2): 316–333.

[62] Camacho M, Alsford S, Jones A, Agnew A (1995) Nicotinic acetylcholine receptors on the surface of the blood fluke Schistosoma. Mol Biochem Parasitol 71: 127–1. 7630376 10.1016/0166-6851(94)00039-p

[63] Camacho M, Agnew A (1995) *Schistosoma*: rate of glucose transport is altered by acetylcholine interaction with tegumental acetylcholine receptors and acetylcholinesterase. Exp Parasitol 81: 584–591. 8543000 10.1006/expr.1995.1152

[64] Kruger FJ, Hamilton-Attwell VL, Tiedt L, Du Preez L (1986) Further observations on an intratubercular sensory receptor of *Schistosoma mattheei.* . Onderstepoort J Vet Res 53(4): 239–40. 3796951

[65] Bentley GN, Jones AK, Oliveros Parra WG, Agnew A (2004) ShAR1alpha and ShAR1beta: novel putative nicotinic acetylcholine receptor subunits from the platyhelminth blood fluke *Schistosoma* . Gene 329: 27–38. 15033526 10.1016/j.gene.2003.12.009

[66] Hamdan FF, Mousa A, Ribeiro P (2002) Codon optimization improves heterologous expression of a *Schistosoma mansoni* cDNA in HEK-293 cells. Parasitol Res 88(6): 583–6. 12107483 10.1007/s00436-001-0585-0

[67] García-Guzmán M, Sala F, Sala S, Campos-Caro A, Criado M (1994) Role of two acetylcholine receptor subunit domains in homomer formation and intersubunit recognition, as revealed by alpha 3 and alpha 7 subunit chimeras. Biochem 33(50): 15198–203. 7999780 10.1021/bi00254a031

[68] Raymond V, Mongan NP, Sattelle DB (2000) Anthelmintic actions on homomer-forming nicotinic acetylcholine receptor subunits: chicken alpha7 and ACR-16 from the nematode *Caenorhabditis elegans* . Neurosci 101(3): 785–91. 10.1016/s0306-4522(00)00279-7 11113327

[69] Le Novère N, Corringer PJ, Changeux JP (2002) The diversity of subunit composition in nAChRs: evolutionary origins, physiologic and pharmacologic consequences. J Neurobiol 53(4): 447–56. 12436412 10.1002/neu.10153

[70] Papke RL, Dwoskin LP (2007) Crooks (2007) The pharmacological activity of nicotine and nornicotine on nAChRs subtypes: relevance to nicotine dependence and drug discovery. J Neurochem 101(1): 160–7. 17241116 10.1111/j.1471-4159.2006.04355.x

[71] Williamson SM, Robertson AP, Brown L, Williams T, Woods DJ, Martin RJ, Sattelle DB, Wolstenholme AJ (2009) The nicotinic acetylcholine receptors of the parasitic nematode *Ascaris suum*: formation of two distinct drug targets by varying the relative expression levels of two subunits. PLoS Pathog 5(7): e1000517. 19609360 10.1371/journal.ppat.1000517 PMC2705655

[72] Colquhoun L, Holden-Dye L, Walker RJ (1991) The pharmacology of cholinoceptors on the somatic muscle cells of the parasitic nematode *Ascaris suum* . J Exp Biol 158: 509–530. 1919416 10.1242/jeb.158.1.509

[73] Trivedi S, Liu J, Ruifeng L, Bostwick R (2010) Advances in functional assays for high-throughput screening of ion channels targets. Expert Opin Drug Discov 5(10): 995–1006. 22823991 10.1517/17460441.2010.513377

[74] Patocka N, Sharma N, Rashid M, Ribeiro P (2014) Serotonin Signaling in *Schistosoma mansoni*: A Serotonin–Activated G Protein-Coupled Receptor Controls Parasite Movement. PLoS Pathog 10(1): e1003878. 24453972 10.1371/journal.ppat.1003878 PMC3894222

