# Supplementary figures and images for: Functional Characterization of a Novel Family of Acetylcholine-Gated Chloride Channels in Schistosoma mansoni
Source: PLoS Pathog. 2014 Jun 12;10(6):e1004181. doi: 10.1371/journal.ppat.1004181 (PMC4055736; doi:10.1371/journal.ppat.1004181)

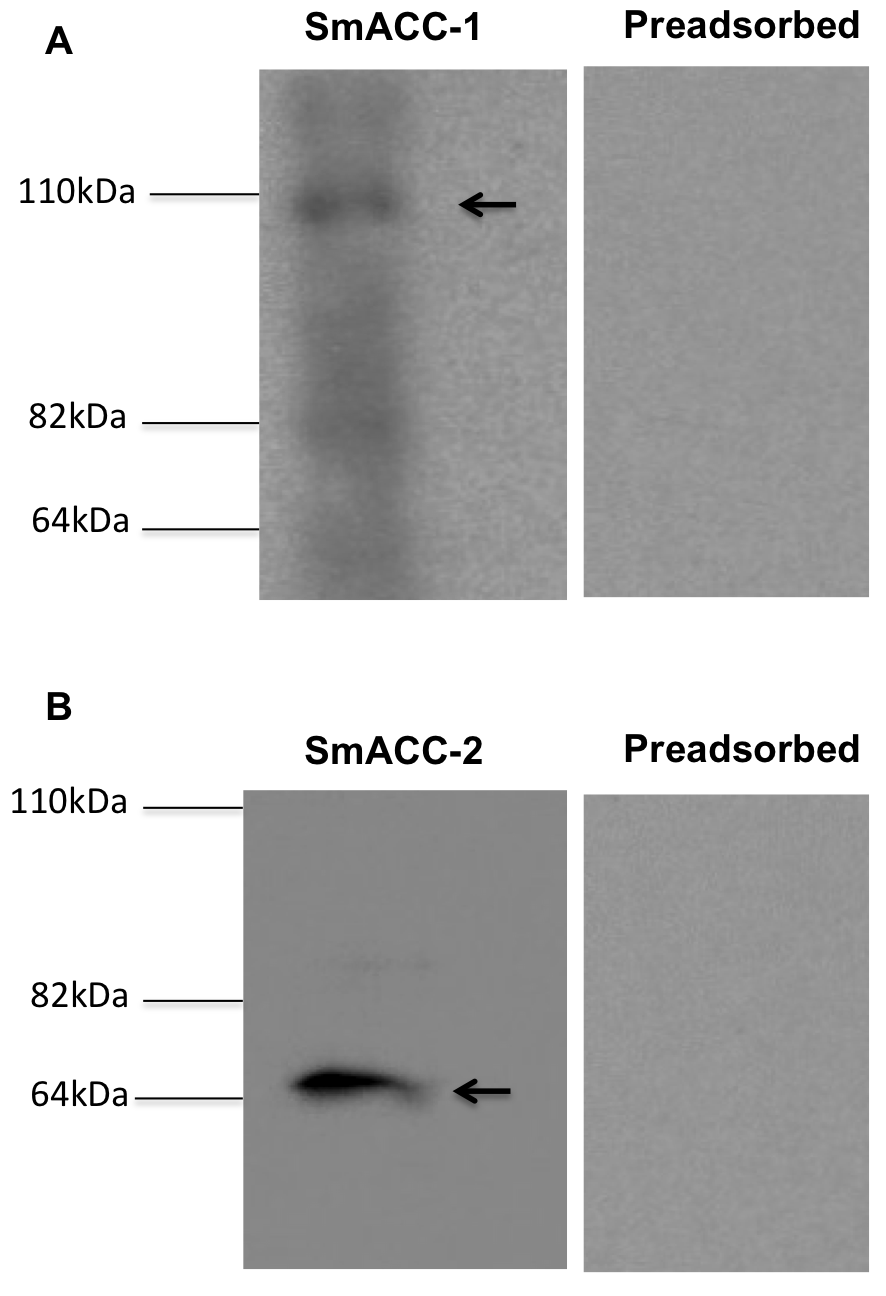

Supplement: Figure S1 — Validation of anti-SmACC antibodies in adult schistosomes. Crude membrane protein extract from adult S. mansoni was run on an SDS-PAGE gel, transferred to a PVDF membrane and probed with affinity-purified anti-SmACC-1 antibody (A) or anti-SmACC-2 antibody (B), followed by horseradish peroxidase (HRP) conjugated secondary antibody. The positions of the two immunoreactive bands are indicated. There was no immunoreactivity in the antigen-preadsorbed negative control for either antibody. (TIF) [file ppat.1004181.s001.tif]
